# Glycated hemoglobin (A1c) values and antidiabetic treatment deprescription in hospitalized nonagenarians with type 2 diabetes

**DOI:** 10.3389/fpubh.2026.1900588

**Published:** 2026-09-09

**Authors:** Elly Morros-González, Paola Andrea Triviño-Beleño, Emily Sharlot Villamizar, Nicolas Torres-Arias, Ángela Marriaga-Ángel

**Affiliations:** 1 Departamento de Clínicas Médicas, Instituto Méderi del Envejecimiento y Longevidad, Hospital Universitario Mayor-Méderi, Universidad del Rosario, Bogotá, Colombia; 2 Facultad de Medicina, Universidad El Bosque, Bogotá, Colombia; 3 Facultad de Ciencias de la Salud de la Universidad Autónoma de Bucaramanga, Bucaramanga, Colombia; 4 Departamento de Clínicas Médicas, Hospital Universitario Mayor-Méderi, Universidad del Rosario, Bogotá, Colombia

**Keywords:** Colombia, deprescription, frailty, glycated hemoglobin, hospitalization, nonagenarian, type 2 diabetes

## Abstract

**Introduction:**

Type 2 diabetes mellitus is highly prevalent among older adults and associated with frailty, functional decline, and increased healthcare utilization. Evidence on metabolic control and therapeutic management in hospitalized nonagenarians remains scarce, particularly in low- and middle-income country settings. This study aimed to describe glycated hemoglobin (A1c) and antidiabetic treatment modifications during hospitalization in nonagenarians with type 2 diabetes, within the context of their clinical, functional, and geriatric characteristics.

**Materials and methods:**

A descriptive, retrospective, cross-sectional study based on an institutional registry from the geriatric service of a high-complexity hospital in Bogotá, Colombia (2022–2024). Individuals aged ≥90 years with a history of type 2 diabetes mellitus documented at admission were included. A1c values, antidiabetic treatment at admission and discharge and geriatric characteristics were analyzed descriptively.

**Results:**

Among 195 nonagenarians, mean age was 92 ± 2 years and 60.00% were women. Infectious conditions were the most frequent admission diagnoses. Median Barthel Index score was 55 points (IQR 20–80); frailty (CFS ≥ 5) was identified in 89.20%, delirium at admission in 39.49%, and major neurocognitive disorder in 16.41%. Of patients with formal nutritional assessment (*n* = 103), 52.43% had moderate-to-severe malnutrition. Polypharmacy was present in 80.00%, with a median of 7 medications (IQR 5–9). Despite this clinical burden, 74.38% of patients with available A1c (*n* = 121) had values <7.5%, suggesting potential overtreatment. At discharge, the proportion without antidiabetic pharmacological treatment increased from 26.15 to 46.20%, with reductions in metformin, DPP-4 inhibitors, and insulin prescriptions, and an increase in SGLT-2 inhibitor use.

**Conclusion:**

The coexistence of low A1c values and high burden of frailty, malnutrition, and polypharmacy reveals a critical discordance between therapeutic intensity and clinical status. These findings highlight a gap in ambulatory therapeutic review and support the need for individualized goals, deprescription protocols, and interdisciplinary care models bridging hospital, community, and domiciliary settings, a public health priority for aging populations in Latin America.

## 1 Introduction

Type 2 diabetes mellitus is a highly prevalent disease in the older adult population. According to data from the International Diabetes Federation, the estimated global prevalence in 2024 among adults aged 20–79 years was 11.1%, while in the population aged 65 to 99 years it was 23.7%. Regarding mortality, in 2024, 63% of deaths attributed to diabetes occurred in individuals over 60 years of age (1). In Latin America and the Caribbean, community-based studies including individuals aged 60 years or older report prevalence ranging from 13% in Santiago de Chile to 22% in Mexico City and Bridgetown (2). In Colombia, the SABE study (2015) reported a prevalence of 18.5% (3), and 17.5% specifically for the city of Bogotá (4).

The growing prevalence of diabetes among older adults is driven by two converging factors: physiological changes associated with aging and increasing life expectancy. Aging is accompanied by pancreatic beta-cell dysfunction, altered hormonal regulation, and progressive changes in body composition that promote insulin resistance, a pro-inflammatory state and a catabolic state with progressive muscle loss (5–7), predisposing older adults to micro- and macrovascular complications, frailty, sarcopenia, polypharmacy, disability, cognitive impairment, infections, and malnutrition (6), predisposing patients to greater care needs and increased costs for health systems. In this context, three variables are of particular clinical relevance: glycemic control, assessed through glycated hemoglobin (A1c) and guiding therapeutic targets and deprescribing decisions (8–10), frailty, and functional dependence for activities of daily living, both of which directly condition therapeutic decision-making and glycemic target individualization in this population (11–13).

Despite the high prevalence of diabetes in older adults, specific data on the clinical profile, metabolic control, and therapeutic management of nonagenarians in the hospital setting remain scarce. Diabetes is associated with a hospitalization rate up to three times higher in adults over 65 compared to those under 45 (14). Cardiovascular disease, chronic kidney disease, and infections represent the leading causes of acute admission in older people with diabetes, which increases infectious susceptibility through hyperglycemia-induced immune dysfunction, peripheral neuropathy, and tissue ischemia (15, 16). In nonagenarians specifically, studies describe infectious conditions, particularly pneumonia and urinary tract infection, as the predominant causes of acute admission, followed by cardiovascular decompensation, a pattern consistent with the extreme frailty and immune vulnerability of this age group (17, 18). Geriatric syndromes including frailty, functional dependence, cognitive impairment, and polypharmacy act as determinants of hospitalization risk and in-hospital prognosis, length of stay, and post-discharge mortality (19–21). Regarding acute diabetic decompensation as a specific cause of hospitalization, hypoglycemia predominates over hyperglycemia in individuals aged ≥75 years, driven by insulin use, chronic kidney disease, cognitive impairment, and prolonged diabetes duration (22–24). Hyperosmolar states tend to be more frequent than isolated diabetic ketoacidosis, as older adults are more susceptible to dehydration and acute kidney injury (25).

Despite population aging, literature on hospitalized patients aged ≥90 years with diabetes remains limited. This population carries a high burden of frailty, comorbidity, and polypharmacy (26), which may complicate glycemic management and increase the risk of inappropriate prescribing and drug–drug or drug–food interactions, among others (27). The pharmacological management of T2D in older adults encompasses oral agents and insulin, each with distinct risk–benefit profiles requiring individualized assessment. Metformin remains a safety option when renal function allows; DPP-4 inhibitors offer a favorable safety profile with low hypoglycemia risk and good tolerability in frail patients. SGLT-2 inhibitors provide cardiorenal benefits but require careful assessment of volume status, renal function, and nutritional state. GLP-1 receptor agonists offer cardiorenal benefits as well, though their use requires monitoring for appetite suppression and lean mass loss, demanding concomitant nutritional support and exercise for sarcopenia prevention (28, 29). Sulfonylureas are generally avoided due to high hypoglycemia risk in patients with frailty or cognitive impairment. Insulin allows flexible glycemic control but is associated with hypoglycemia, falls, weight gain, and increased treatment burden, often requiring caregiver assistance for administration in patients with functional dependence or cognitive impairment (8, 10, 30), risks that are amplified in nonagenarians with polypharmacy, renal impairment, and irregular nutritional intake (8, 29, 30). Drug–drug interactions, particularly between insulin or sulfonylureas and medications that potentiate hypoglycemia, including beta-blockers, ACE inhibitors, and fluoroquinolones or those that alter renal clearance of antidiabetic agents (30).

In nonagenarians, multimorbidity drives the high pharmacological burden characteristic of this age group. Polypharmacy, conventionally defined as the concurrent use of five or more medications, is highly prevalent in hospitalized older adults and is independently associated with adverse drug reactions, drug–drug interactions, falls, delirium, and functional decline (31). Beyond conventional polypharmacy, the concept of hidden polypharmacy refers to the overlapping pharmacokinetic and pharmacodynamic mechanisms of drugs acting on shared targets, such as biguanides, statins, and serotonergic agents, which may generate interactions and adverse effects even when the total number of medications appears acceptable (32). Both forms of polypharmacy increase the risk of hypoglycemia, renal impairment, and cardiovascular events. These considerations underscore the need for individualized therapeutic goals and periodic medication review guided by comprehensive geriatric assessment, to optimize glycemic management and minimize inappropriate prescribing and its associated risks.

In fragmented health systems such as those of Colombia and Latin America, care for older adults with diabetes frequently defaults to the hospital setting, reflecting gaps in primary care’s capacity for individualized management, limited access to specialists, and insufficient interdisciplinary care encompassing education, nutrition, and targeted physical activity (33), as well as difficulties in ensuring coordination across levels of care. On the other hand, weak health literacy in communities acts as a barrier and is associated with poor glycemic control, reduced medication adherence, and higher hospitalization rates; this particularly prevalent in older adults with low educational attainment, cognitive impairment, or limited social support (9, 34). Strengthening health literacy through tailored education, simplified care plans, and caregiver involvement is a key public health strategy to complement clinical management and reduce preventable complications in this population.

Evidence to guide decisions in hospitalized nonagenarians is scarce, limiting support for clinical and public health decision-making in this age group. Therefore, the objective of this study was to describe A1c values and antidiabetic treatment modifications during hospitalization in patients aged ≥90 years with type 2 diabetes, within the context of their clinical, functional, and geriatric characteristics.

## 2 Materials and methods

A retrospective descriptive study was conducted based on an institutional registry of the geriatrics department of a high-complexity hospital (AGING – Atención Geriátrica INtegral), which serves patients covered under both contributory and subsidized health insurance schemes. All patients aged 90 years or older admitted to the geriatrics service between November 1, 2022, and February 29, 2024, with a history of type 2 diabetes mellitus at admission (by self-report or clinical record) were eligible for inclusion. Data on antidiabetic treatment at admission and discharge, and glycated hemoglobin (A1c), were retrieved from the hospital’s electronic medical records system. Patients referred from other institutions and those whose hospitalization was not completed at the institution due to administrative transfer were excluded, as comprehensive geriatric care and adverse event monitoring could not be guaranteed in other settings. All eligible patients meeting inclusion criteria during the study period were consecutively enrolled; no probabilistic sampling or sample size calculation was performed, as the study aimed to describe the complete population of eligible patients within the defined timeframe.

The AGING registry contains information on patients consecutively admitted to the geriatrics service since 2022, for both clinical follow-up and research purposes, and is funded by the Méderi Research Center (CIMED). All variables are derived from the comprehensive geriatric assessment (CGA) performed at hospital admission by geriatricians, reflecting the patient’s baseline status in the 15 days prior to the onset of the condition leading to hospitalization. Data are recorded by a trained research assistant, with periodic audits conducted by both CIMED and the research group.

Through the comprehensive geriatric assessment performed from the time of admission, which encompasses clinical, cognitive, nutritional, functional, and social domains, individualized management objectives and goals are established for each patient (35). During the hospital stay, medication prescriptions are reviewed daily, with assessment of potential side effects and adverse drug reactions. Prior to discharge, medication reconciliation and screening for inappropriate prescribing are routinely performed.

### 2.1 Variables of interest

Sociodemographic variables collected at admission included: age, sex, educational level (none, primary, secondary, university), marital status (single, married, widowed, separated), living arrangement (alone, with spouse, with children, institutionalized), and place of residence (urban/rural).

Clinical and geriatric variables included comorbidities, reason for hospitalization, anthropometric measurements, and pharmacological burden. Polypharmacy was defined as the use of ≥5 medications (36).

Baseline status was assessed across functional, frailty, cognitive, and social domains. Functional status was evaluated using the Barthel Index for basic activities of daily living (independence ≥90 points; mild dependence 60–85; moderate 40–55; severe 20–35; total dependence ≤15) (12, 37) and the Lawton Instrumental Activities of Daily Living Scale (increasing dependence from 8 to 0 points) (13). Frailty was assessed using the Clinical Frailty Scale (CFS), with scores ≥5 indicating frailty (11, 38). Geriatric syndromes were documented, including falls in the past year (recurrent: ≥2), delirium at admission (assessed by the Confusion Assessment Method [CAM] ≥ 3 or clinical diagnosis recorded in the medical chart) (39, 40), and nutritional status using the Mini Nutritional Assessment–Short Form (MNA-SF) (malnutrition 0–7; at risk 8–11; adequate ≥12) (41).

Regarding diabetes-specific pharmacological management, antidiabetic medications were recorded at admission based on patient or caregiver self-report as documented in the medical chart, and at discharge based on the final clinical note. A1c was analyzed from measurement taken during hospitalization, or within the preceding three months. Glycemic variability through point-of-care glucose monitoring or advanced devices was not assessed, as this information was unavailable.

Deprescribing was operationally defined as the intentional discontinuation or dose reduction of one or more antidiabetic medications at hospital discharge (42, 43), guided by the findings of the comprehensive geriatric assessment, which informed individualized glycemic targets according to the patient’s functional status, frailty level, nutritional status, and risk of hypoglycemia. Medication reconciliation and screening for potentially inappropriate prescribing were conducted prior to discharge, using internationally validated tools including the Screening Tool of Older Persons’ Prescriptions/Screening Tool to Alert to Right Treatment (STOPP/START) (44) criteria and the American Geriatrics Society (AGS) Beers Criteria (45) as reference frameworks. Treatment simplification and deprescribing were considered when strict glycemic targets (A1c < 7.5%) were judged discordant with the patient’s functional status, frailty level, life expectancy, and risk of hypoglycemia, in accordance with current guidelines for diabetes management in older adults (8, 10).

During hospitalization, clinical nutrition assessment, nutritional supplementation requirements, physical therapy evaluation, and the occurrence of adverse events were recorded.

### 2.2 Statistical analysis

A descriptive analysis of all variables was performed according to their nature. Continuous variables were summarized using mean and standard deviation or median and interquartile range, depending on their distribution; categorical variables were reported as absolute and relative frequencies. The point prevalence (proportion) of glycemic control was estimated as the number of patients with A1c < 7.5% divided by the total number of patients with available A1c measurements. Variables with missing data were not imputed; analyses involving those variables were performed on the subset of patients with available information, and the number of valid observations is reported for each variable.

As an exploratory analysis, stratified descriptive analyses were conducted to examine: the distribution of functional dependence (Barthel Index) and frailty (CFS) according to polypharmacy status (≥5 medications); and the distribution of glycemic control (A1c) according to nutritional status (MNA-SF). Continuous variables were compared using the Mann–Whitney U test and are presented as median (interquartile range). Categorical variables were compared using the chi-square test or Fisher’s exact test as appropriate, and are presented as absolute frequencies and percentages. These analyses are hypothesis-generating only and do not imply causal inference.

Descriptive graphs were constructed to illustrate the distribution of A1c values and changes in antidiabetic treatment between admission and discharge. All analyses were performed in RStudio version 4.2.2.

### 2.3 Ethical considerations

This study was classified as no-risk research according to national regulations (Resolution 8430/1993) and was conducted in accordance with the Declaration of Helsinki. It was approved by the Research Ethics Committee of Universidad del Rosario, with waiver of informed consent (DVO005 2,865-CV1879). Data were obtained from the institutional AGING registry, which holds independent ethics approval (CEISH-2025002).

## 3 Results

### 3.1 General, clinical, and baseline characteristics

**Figure 1:**
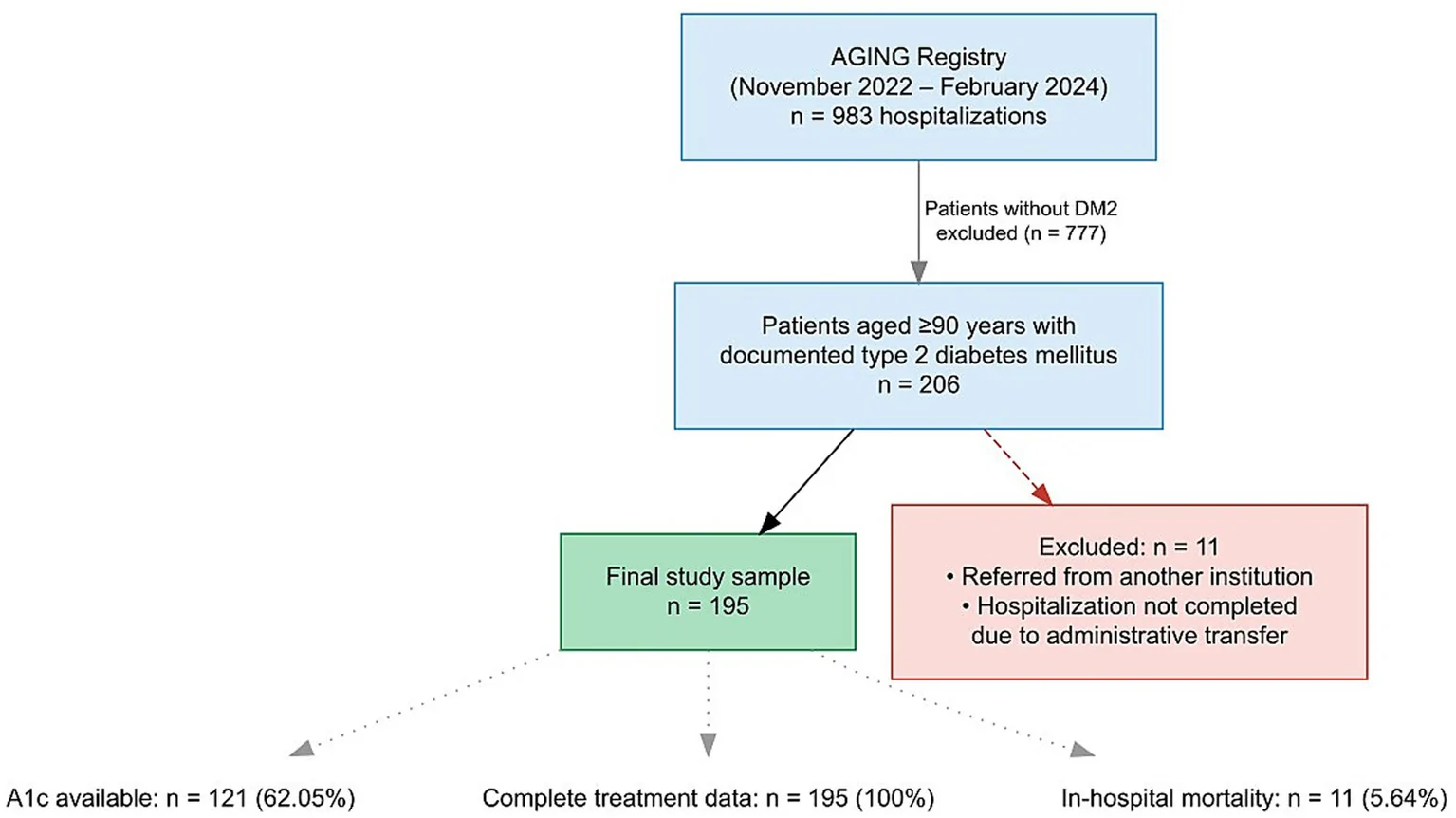
Patient selection flowchart.

According to the registry, 195 nonagenarians had a history of type 2 diabetes mellitus (see Figure 1). The mean age was 92 years (±2.35 years); most patients were urban residents, with a higher proportion of women than men. A total of 122 (62.60%) had completed primary school as their highest level of education, and 65.64% were widowed. Among participants, 74.34% lived with their children and 18.46% with their partner.

Regarding clinical history, 66 (33.85%) had been previously hospitalized within the past year. The most frequent reasons for admission were pneumonia (30, 15.38%) and urinary tract infection (34, 17.44%), followed by decompensated heart failure (15, 7.59%). The most prevalent comorbidity was hypertension, present in 171(87.69%) of patients, followed by COPD in 68 (34.87%) and hypothyroidism in 64 (32.82%) (see Table 1). Polypharmacy was present in 156 (80.00%) of patients, with a median of 7 medications per patient; biguanides and dipeptidyl peptidase-4 inhibitors (DPP-4i) were the most frequently antidiabetic agents prescribed before hospital admission.

**Table 1:** General, clinical, and baseline characteristics.

Variable		Total = 195 *n* (%)
Sociodemographic		
Sex	Female	117 (60.00%)
Age	92 ± 2 years min 90, max 101	
Residential area	Urban	189 (95.90%)
Education	Primary school	122 (62.56%)
	High school / technician	34 (17.44%)
	University	4 (2.05%)
	None	24 (12.30%)
	No information	11 (5.65%)
Clinical		
Comorbidities	Hypertension	171 (87.69%)
	COPD	68 (34.87%)
	Hypothyroidism	64 (32.82%)
	Chronic kidney disease	41 (21.03%)
	Neurocognitive disorder	32 (16.41%)
	Cerebrovascular disease	22 (11.28%)
Medications at admission	Polypharmacy	156 (80.00%)
	Median 7 (IQR 5–9) min 0, max 18	
BMI (kg/m2) *n* = 195	Median 24.10 min 15, max 39.50	
	<22	64 (32.82%)
	22–28	92 (47.18%)
	>28	39 (20.00%)
MNA-SF	Median 9 (IQR 7–11)	
Baseline status		
Barthel Index	Median 55 (IQR 20–80)	
	Independent	31 (15.90%)
	Mild dependence	62 (31.80%)
	Moderate dependence	28 (14.36%)
	Severe dependence	30 (15.38%)
	Total dependence	44 (22.56%)
Clinical Frailty Scale CFS *n* = 176	CFS ≥ 5	157 (89.20%)
Falls in the past year	Yes	61 (31.28%)
	Recurrent falls	34 (55.73%)
Delirium at admission	Yes	77 (39.49%)
Type of major neurocognitive disorder *n* = 32	Alzheimer’s disease	18 (56.25%)
	Mixed (vascular and Alzheimer’s)	2 (6.25%)
	Vascular	2 (6.25%)
	Unspecified	10 (31.25%)
Severity of neurocognitive disorder *n* = 32	Mild	0
	Moderate	5 (15.62%)
	Severe	17 (53.13%)
	Not reported	10 (31.25%)

COPD, chronic obstructive pulmonary disease; BMI, body mass index; MNA-SF, mini nutritional assessment–short form; CFS, Clinical Frailty Scale; IQR, interquartile range.

Functional status for basic activities of daily living had a median Barthel Index score of 55 (IQR 20–80). A total of 132 (67.69%) were fully dependent for instrumental activities of daily living according to the Lawton and Brody Scale. Frailty by deficit accumulation, defined as a CFS score ≥5, was present in 157 (89.20%) of patients. Falls in the past year were frequent, occurring in 61 (31.28%) of patients. Delirium at admission was identified in 77 (39.49%), and a diagnosis of major neurocognitive disorder was present in 32 (16.41%), with Alzheimer’s disease being the most common etiology, predominantly at a severe stage.

### 3.2 In-hospital characteristics

A total of 103 (52.28%) patients received a formal clinical nutrition assessment; of these, 54 (52.43%) were diagnosed with moderate-to-severe malnutrition, while 13 (12.62%) had overweight or obesity, and 47 (45.63%) received nutritional supplementation (see Table 2). Physical therapy evaluation was conducted in 85 (43.59%) of patients, and 18 (9.23%) developed in-hospital delirium.

**Table 2:** In-hospital and discharge variables.

Variable		Total = 195 *n* (%)
Admission diagnosis	Pneumonia	30 (15.38%)
	Urinary tract infection	34 (17.44%)
	Decompensated heart failure	15 (7.69%)
	Upper gastrointestinal bleeding	9 (4.62%)
Clinical nutrition assessment and diagnosis *n* = 103 (52.82%)	Moderate-to-severe malnutrition	54 (52.43%)
	Risk of malnutrition	23 (22.33%)
	Well-nourished	13 (12.62%)
	Overweight–obesity	13 (12.62%)
Nutritional supplementation required	Yes	47 (45.63%)
Physical therapy assessment	Yes	85 (43.59%)
In-hospital delirium	Yes	18 (9.23%)
Adverse event during hospitalization	Yes	8 (4.10%)
	Bacteriemia	4 (50.00%)
	Pneumonia	3 (37.50%)
	Pressure ulcers	1 (12.50%)
	Other	1 (12.50%)
Discharge reason	Planned intervention completed	109 (55.89%)
	Extended care or home-based care	73 (37.44%)
	Death	11 (5.64%)
	Voluntary discharge	2 (1.03%)
Length of hospital stay	Median 6 (IQR 4–9) min 1, max 53	

IQR, interquartile range.

The median length of hospital stay was 6 days (IQR 4–9 days). 8 patients (4.06%) experienced adverse events, including bacteremia in 4 cases and healthcare-associated pneumonia in 3 patients. In-hospital management was completed in 109 (55.89%) of patients, while 73 (37.44%) were discharged to extramural care, and the in-hospital mortality frequency was 5.64% (11 patients). Among the 11 patients who died, women predominated (7, 63.63%), and all presented with delirium at admission and frailty (CFS ≥ 5). Total functional dependence was the most frequent category (9, 81.81%), with a median CFS of 7 (IQR 7–8). Polypharmacy was present in 9 patients (81.81%), with a median hospital stay of 8 days (IQR 4.5–9.0). The most frequent comorbidities were hypertension (8, 72.72%), COPD (7, 63.63%), and hypothyroidism (5, 45.45%). The predominant admission diagnosis was pneumonia (4, 36.36%), followed by urinary tract infection, soft tissue infection, and upper gastrointestinal bleeding. A1c was available in 4 patients, with a median of 7.65% (IQR 6.57–8.50).

### 3.3 Metabolic control and deprescribing

**Figure 2:**
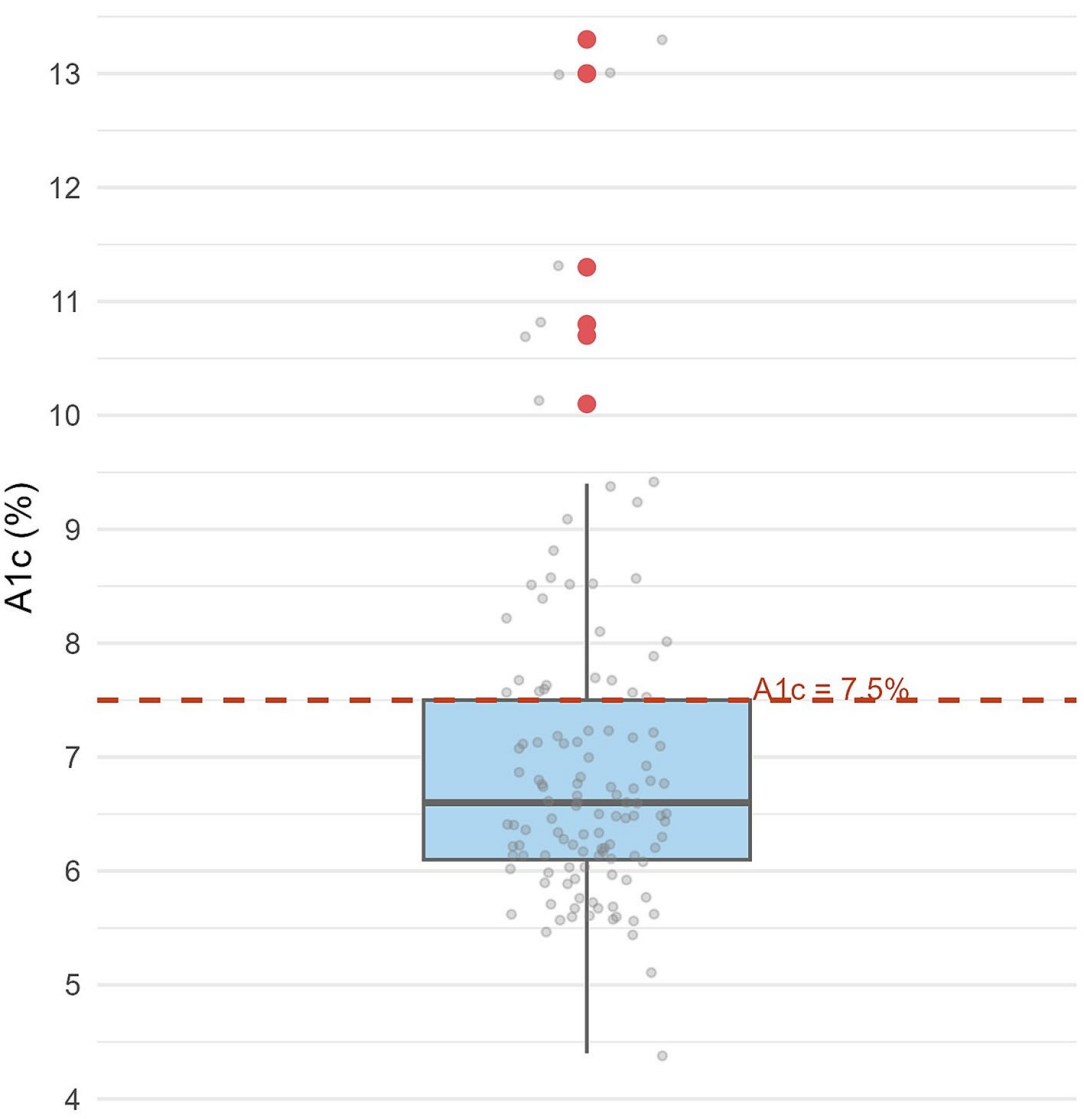
Box-and-whisker plot showing the distribution of glycated hemoglobin (A1c, %) values.

**Figure 3:**
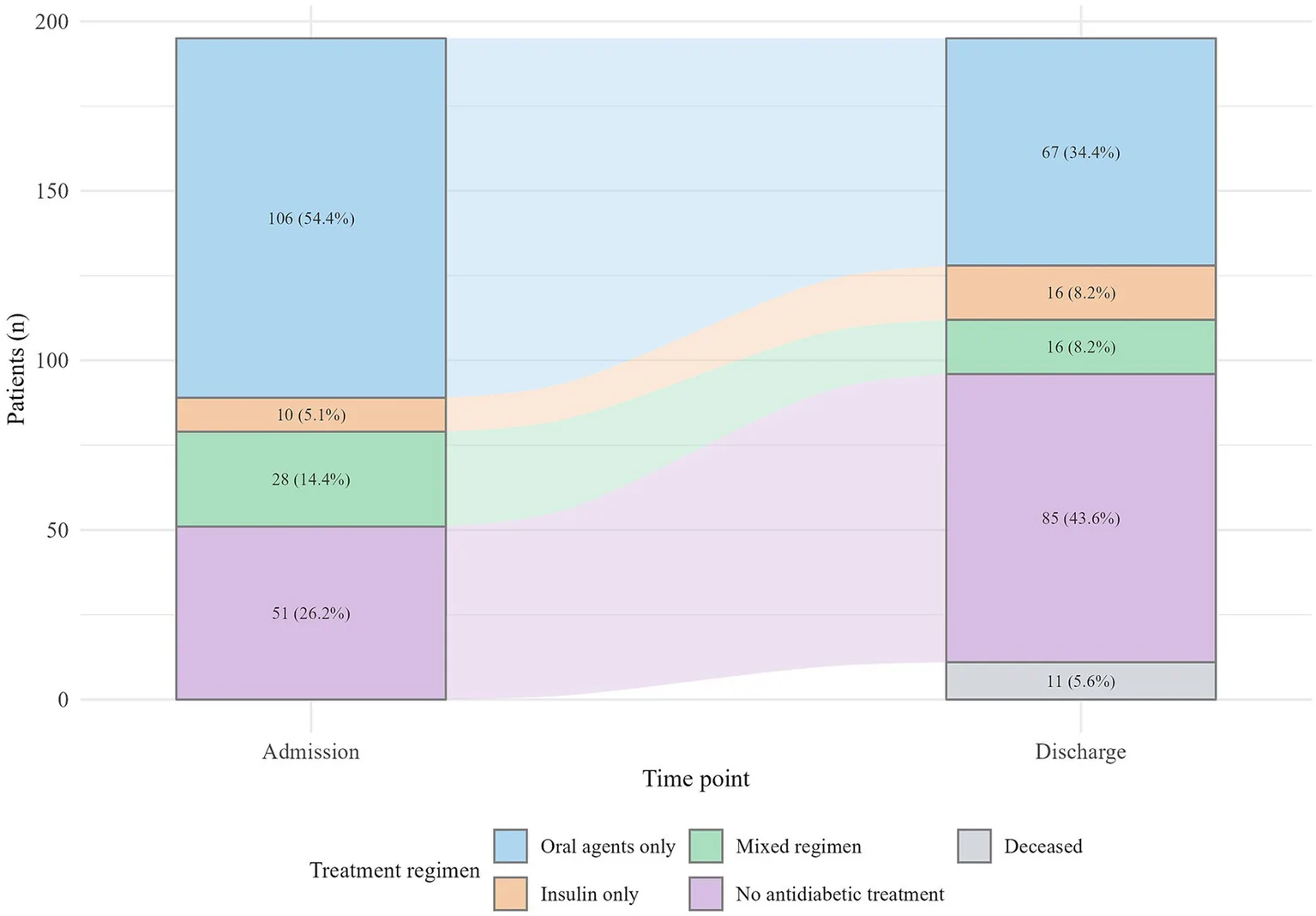
Patterns of antidiabetic treatment modification during hospitalization in nonagenarians with type 2 diabetes (*n* = 195).

**Figure 4:**
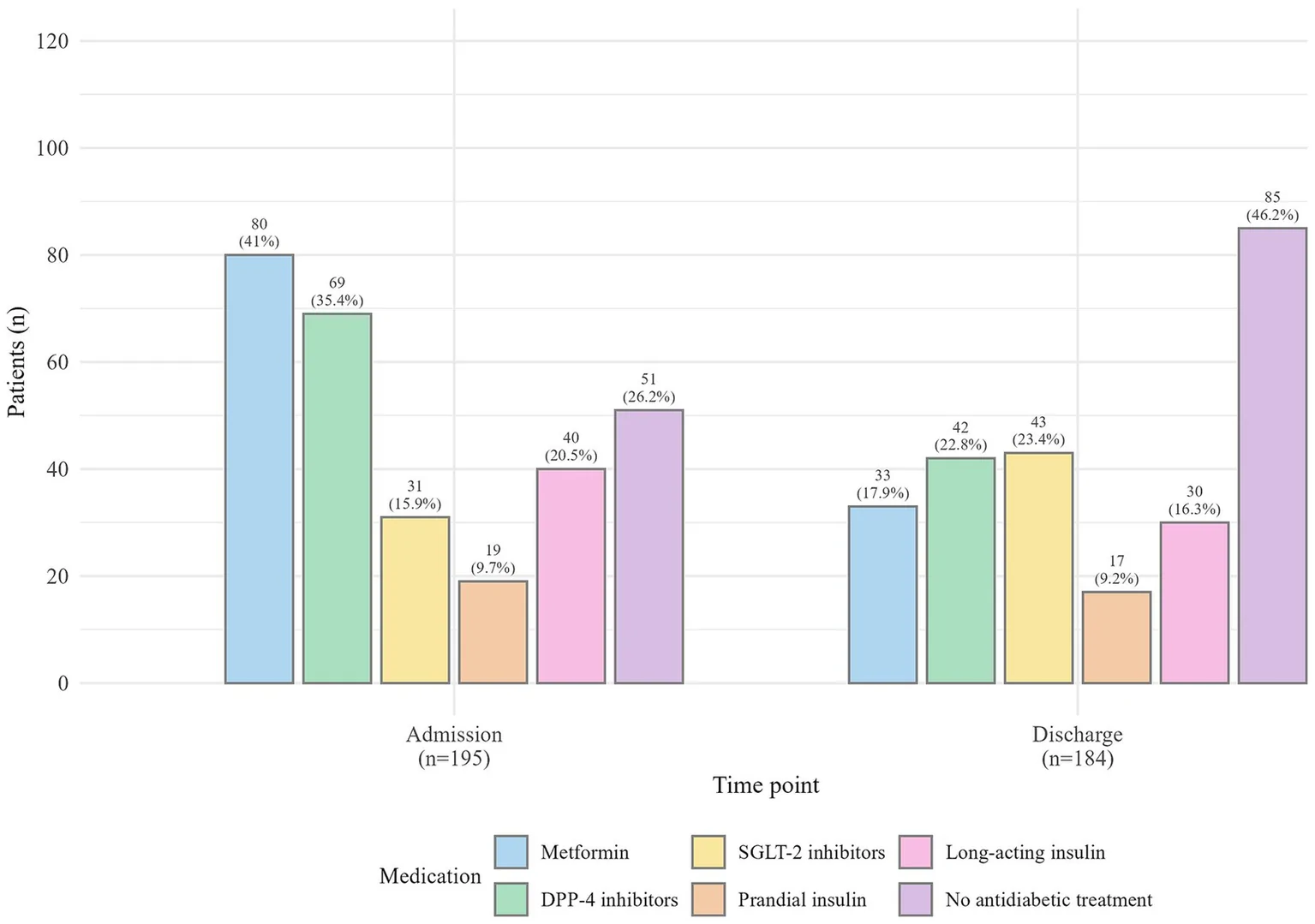
Changes in antidiabetic medication use at admission (*n* = 195) and discharge (*n* = 184).

Regarding metabolic control (*n* = 121), 90 (74.38%) of patients had an A1c < 7.5%; the distribution of A1c values in this sample is shown in Figure 2. At discharge, among 184 patients who survived hospitalization, 85 (46.20%) required no antidiabetic pharmacological treatment, compared to 51 (26.15%) at admission. Metformin prescription decreased from 80/195 (41.03%) to 33/184 (17.93%), DPP-4i from 69/195 (35.38%) to 42/184 (22.83%), long-acting insulin from 40/195 (20.51%) to 30/184 (16.30%), and prandial insulin from 19/195 (9.74%) to 17/184 (9.24%). SGLT-2 inhibitor prescriptions increased from 31/195 (15.89%) to 43/184 (23.37%). Overall patterns of antidiabetic treatment regimen modification are shown in Figure 3, and changes by individual agent in Figure 4.

### 3.4 Exploratory analysis

In the exploratory stratified analysis, no statistically significant differences in A1c values were observed between patients with malnutrition or nutritional risk and those who were well-nourished (A1c < 7.5%: 41 (74.5%) vs. 19 (79.2%), χ^2^ = 0.024, df = 1, *p* = 0.876).

The distribution of functional dependence categories differed significantly between patients with and without polypharmacy (χ^2^ = 13.146, df = 4, *p* = 0.011), with a higher proportion of mild dependence among those with polypharmacy (56, 35.9%) compared to those without (6, 15.4%) and a higher proportion of independence among those with polypharmacy (19, 12.2%) versus those without (12, 30.8%). No statistically significant differences were observed in frailty CFS score (Mann–Whitney U: W = 2,604.5, *p* = 0.903), Barthel score (Mann–Whitney U: W = 2,997, *p* = 0.888) or A1c values (A1c < 7.5%: 74 (75.5%) vs. 16 (69.6%); χ^2^ = 0.104, df = 1, *p* = 0.747) between patients with and without polypharmacy.

## 4 Discussion

This study analyzed 195 nonagenarians with type 2 diabetes hospitalized in geriatric service in Bogotá, Colombia. A high burden of frailty, functional dependence, and malnutrition was identified, accompanied by predominantly low A1c values; and a high rate of antidiabetic deprescription at discharge, revealing a discordance between prior therapeutic intensity and clinical status at discharge.

The predominance of women, primary-level education, widowhood and frequent cohabitation with children is consistent with data for older adults in Bogotá, Colombia through the Cuenta de Alto Costo (46). Low educational attainment is likewise consistent with prior studies (4), as it has been associated with reduced capacity for diabetes self-management, lower medication adherence and higher hospitalizations rates (34). These sociodemographic profiles represent social determinants that condition access to care and therapeutic adherence; in fragmented health systems such as Colombia’s, this vulnerability may translate into preventable hospitalizations and reduced continuity of care after discharge (33).

Regarding baseline functional status, the majority of patients had some degree of dependence, with mild functional dependence in 31.97% and total functional dependence in 22.34% for basic activities of daily living. Higher prevalences have been reported in studies conducted in Peru in older adult populations, albeit of younger age (47). Baseline dependence is a well-established risk factor for in-hospital functional decline, higher complications rates, prolonged stay, economic and social costs, resource consumption, readmissions, and increased post-discharge mortality (48). From a geriatric perspective, collaborative work with an interdisciplinary team, including nursing, rehabilitation, nutrition, and social work, is therefore essential for the prevention of immobility-related complications, delirium, nutritional deterioration, and other adverse outcomes. In the Latin American context, the effective implementation of interdisciplinary teams faces significant barriers, including a shortage of geriatric specialists, the absence of standardized functional assessment protocols, and limited integration between hospital services and community or home-based rehabilitation resources, making the resolution of these gaps a health policy priority (33).

Frailty, assessed by CFS prior to the acute condition, was identified in 89.20%, a geriatric syndrome widely described in older adults with diabetes (6). This high prevalence reflects shared pathophysiological pathways between diabetes, aging, frailty, and sarcopenia (5, 7, 49), as well as the extreme age of the population, since age itself has been described as an independent risk factor (50, 51). Prevalence varies across studies depending on the instrument and care setting, ranging from 26% using the FRAIL scale to 36% using the Tilburg Frailty Indicator (50). These findings reinforce the need for systematic frailty screening across all care levels, as a criterion for individualizing glycemic targets, consistent with current guidelines (8, 10, 15), and for strengthening primary care competencies in early frailty identification.

Regarding nutritional status, moderate-to-severe malnutrition was identified in 52.43% of patients receiving formal nutritional assessment, consistent with estimates in hospitalized older adults (52), and polypharmacy was present in 80.00%, with median of 7 medications, higher than rates reported in comparable populations (53), likely reflecting the multimorbidity burden of nonagenarians (29, 30) and high risk of pharmacological interactions with a subsequent increased risk of hypoglycemia, falls, syncope, prolonged hospitalization, and death (30, 52, 53). In the exploratory analysis, the distribution of functional dependence categories differed significantly between patients with and without polypharmacy (*p* = 0.011), with a higher proportion of mild dependence among those with polypharmacy (35.9% vs. 15.4%) and greater independence among those without (30.8% vs. 12.2%), while no significant differences were observed in frailty severity (*p* = 0.763). Regarding nutritional status, no statistically significant differences in A1c values were found between patients with malnutrition or nutritional risk and those who were well-nourished (A1c < 7.5%: 74.5% vs. 79.2%, *p* = 0.876). Both analyses are exploratory and should be interpreted as hypothesis-generating signals only.

Despite the high burden of frailty, functional dependence, malnutrition, and polypharmacy, 74.38% of patients with available A1c had values <7.5%, suggesting potential overtreatment relative to their clinical status. Current guidelines recommend less stringent glycemic targets, such as A1c 7.5–8.5% for older adults with frailty, functional dependence, or high hypoglycemia risk (8, 10, 54). This discordance likely reflects therapeutic inertia and the persistence of population-level targets that do not account for the heterogeneity of extreme aging (51), constituting real-world evidence of glycemic overtreatment with implications for clinical practice guideline development.

At discharge, the proportion of patients without antidiabetic pharmacological treatment increased from 26.15 to 46.20%, with reductions in metformin, DPP-4 inhibitors, and insulin, reflecting systematic application of comprehensive geriatric assessment to therapeutic decision-making (43). The increase in SGLT-2 inhibitor prescriptions (15.89 to 23.37%) was limited to patients with preserved renal function and specific cardiovascular or renal indications, including heart failure management, consistent with evidence supporting their cardiorenal benefits in selected older adults (8, 10, 29). These deprescription rates suggest that hospitalization functioned as a systematic opportunity for therapeutic review in many patients, highlighting a critical gap in ambulatory care continuity.

The in-hospital mortality rate observed in this cohort (5.64%) is notably lower than rates reported in comparable populations. A recent scoping review reported median hospital mortality of 25.55% in nonagenarians, with rates ranging from 35–60% in intensive care unit settings. Studies in very old adults with diabetes suggest that diabetes per se may not independently increase in-hospital mortality, a finding consistent with our results (55). Notably, all patients who died presented with delirium at admission, total or severe functional dependence, and severe frailty, highlighting these as key prognostic markers in this population, consistent with prior literature (19, 21, 56, 57).

From a public health perspective, these findings reveal a systemic gap in the ambulatory review of therapeutic goals for nonagenarians with diabetes in fragmented Latin American health systems (33). Strengthening interdisciplinary care models, improving primary care training in geriatric diabetes management, and adapting clinical practice guidelines to the heterogeneity of extreme aging represent both a clinical priority and a public health imperative for aging populations in Latin America (8, 10).

This study has several limitations that should be considered. First, the cross-sectional, descriptive design based on a single institutional registry does not allow causal inferences between clinical variables, metabolic control, and in-hospital outcomes. Second, a selection bias cannot be excluded, as the registry corresponds to a single high-complexity hospital serving a population with contributory and subsidized insurance coverage; patients included likely carry a higher burden of comorbidity and frailty than those managed at lower-complexity settings, limiting the generalizability of findings to other care levels and contexts within Colombia and Latin America. Third, the diagnosis of type 2 diabetes was based on self-report upon admission, or diagnosis in prior clinical records, which may underestimate the true prevalence of diabetes in this age group. Finally, in-hospital point-of-care glucose monitoring was not available, precluding the characterization of glycemic variability during the hospital stay and A1c was available in 62.05% of patients (121/195), reflecting the retrospective registry-based design. Results from exploratory analyses should be interpreted as hypothesis-generating only.

Among the strengths, to our knowledge, this is the first study in Colombia, and one of very few in Latin America, to describe the clinical profile, metabolic control, and therapeutic changes in hospitalized patients aged ≥90 years with type 2 diabetes, contributing real-world evidence from a context that is underrepresented in the international literature. The use of a prospective registry with standardized comprehensive geriatric assessment, conducted by geriatric specialists and audited periodically, supports the quality and consistency of the data collected.

Taken together, these findings highlight hospitalization as a critical opportunity for individualized therapeutic reassessment in nonagenarians with diabetes, and underscore the need for longitudinal, multicenter studies evaluating integrated care interventions in this population, with emphasis on outcomes such as quality of life, functional status, and adverse events. They also call for strengthening the training of primary care physicians and other specialists in the geriatric management of older adults with diabetes, the development of interdisciplinary care models, and the formulation of clinical practice guidelines and health policies, at local, regional, and global levels, that explicitly account for extreme aging, frailty, and the heterogeneity of the oldest-old.

## 5 Conclusion

In this sample of hospitalized nonagenarians with type 2 diabetes, low A1c values were frequent despite the high burden of frailty, functional dependence, malnutrition, and polypharmacy, revealing a critical discordance between therapeutic intensity and clinical status. Hospitalization, guided by comprehensive geriatric assessment, can represent a critical opportunity for therapeutic reassessment, as supported by the high deprescription rates observed at discharge. Strengthening interdisciplinary care models and adapting clinical guidelines to the heterogeneity of extreme aging, as well as the need for individualized goals and deprescription protocols bridging hospital, community, and domiciliary settings, represents a public health priority for aging populations in Latin America.

## Data Availability

The raw data supporting the conclusions of this article will be made available by the corresponding author on request.
